# Ultrasensitive Electroanalytical Detection of Pb^2+^ and H_2_O_2_ Using Bi and Fe—Based Nanoparticles Embedded into Porous Carbon Xerogel—The Influence of Nanocomposite Pyrolysis Temperatures

**DOI:** 10.3390/gels9110868

**Published:** 2023-10-31

**Authors:** Mihai M. Rusu, Carmen I. Fort, Adriana Vulpoi, Lucian Barbu-Tudoran, Monica Baia, Liviu C. Cotet, Lucian Baia

**Affiliations:** 1 Department of Physics and Chemistry, Technical University of Cluj-Napoca, 400114 Cluj-Napoca, Romania; mihai.rusu@phys.utcluj.ro; 2 Laboratory of Advanced Materials and Applied Technologies, Institute of Research-Development-Innovation in Applied Natural Sciences, “Babes-Bolyai” University, Fantanele 30, 400294 Cluj-Napoca, Romania; monica.baia@ubbcluj.ro (M.B.); cosmin.cotet@ubbcluj.ro (L.C.C.); 3 Department of Chemical Engineering, Faculty of Chemistry and Chemical Engineering, “Babes-Bolyai” University, Arany Janos 11, 400028 Cluj-Napoca, Romania; 4 Nanostructured Materials and Bio-Nano-Interfaces Center, Institute of Interdisciplinary Research in Bio-Nano-Sciences, “Babes-Bolyai” University, T. Laurean 42, 400271 Cluj-Napoca, Romania; adriana.vulpoi@ubbcluj.ro; 5 Electron Microscopy Laboratory “Prof. C. Craciun”, Faculty of Biology and Geology, “Babes-Bolyai” University, Clinicilor Str. 5–7, 400006 Cluj-Napoca, Romania; lucian.barbu@ubbcluj.ro; 6 Electron Microscopy Integrated Laboratory, National Institute for Research and Development of Isotopic and Molecular Technologies, 400293 Cluj-Napoca, Romania; 7 Department of Biomolecular Physics, Faculty of Physics, “Babes-Bolyai” University, M. Kogalniceanu 1, 400084 Cluj-Napoca, Romania; 8 Department of Condensed Matter Physics and Advanced Technologies, Faculty of Physics, “Babes-Bolyai” University, M. Kogalniceanu 1, 400084 Cluj-Napoca, Romania

**Keywords:** carbon xerogel, bismuth nanoparticles, iron nanoparticles, nanocomposites, pyrolytic effects, Pb^2+^ detection, H_2_O_2_ detection

## Abstract

Multifunctional materials based on carbon xerogel (CX) with embedded bismuth (Bi) and iron (Fe) nanoparticles are tested for ultrasensitive amperometric detection of lead cation (Pb^2+^) and hydrogen peroxide (H_2_O_2_). The prepared CXBiFe-T nanocomposites were annealed at different pyrolysis temperatures (T, between 600 and 1050 °C) and characterized by X-ray diffraction (XRD), Raman spectroscopy, N_2_ adsorption, dynamic light scattering (DLS), and electron microscopies (SEM/EDX and TEM). Electrochemical impedance spectroscopy (EIS) and square wave anodic stripping voltammetry (SWV) performed at glassy carbon (GC) electrodes modified with chitosan (Chi)-CXBiFe-T evidenced that GC/Chi-CXBiFe-1050 electrodes exhibit excellent analytical behavior for Pb^2+^ and H_2_O_2_ amperometric detection: high sensitivity for Pb^2+^ (9.2·10^5^ µA/µM) and outstanding limits of detection (97 fM, signal-to-noise ratio 3) for Pb^2+^, and remarkable for H_2_O_2_ (2.51 µM). The notable improvements were found to be favored by the increase in pyrolysis temperature. Multi-scale parameters such as (i) graphitization, densification of carbon support, and oxide nanoparticle reduction and purification were considered key aspects in the correlation between material properties and electrochemical response, followed by other effects such as (ii) average nanoparticle and Voronoi domain dimensions and (iii) average CXBiFe-T aggregate dimension.

## 1. Introduction

The engineering of new nanocomposite materials influences the progress and innovation in various fields of applications such as microfluidic systems, catalysis, lab-on-chip devices, energetics, and sensors [1,2,3]. The growth of metal/oxide nanoparticles on carbon supports such as porous xerogels and aerogels has favorable results for applications such as magnetic adsorbents, filters, energy storage, and electrochemical sensors, i.e., for heavy metal detection [4,5].

Carbon xerogels (CX) and/or aerogels represent a subclass of the carbon family described as a vitreous, electron-conductive network with controllable texture and porosity [6]. In the design of competitive, high-quality carbon-based electrochemical sensors and devices, one challenging aspect is achieving both large active surface areas endowed with high resistance to thermal, mechanical, and chemical stress and high conductivity and interfacial charge transfer properties that directly impact the electrochemical (bio)sensor performances (i.e., sensitivity, low detection limit, and low background current). In this context, recent efforts have been directed towards the formation of graphitized nanostructures during the pyrolysis step through heterogeneous graphitization in the presence of embedded transition metal nanoparticles such as Cr, Fe, Co, Ni [7,8], etc. Among these, the Fe-based nanoparticles not only served as graphitizing catalysts or magnetic centers but also allowed the electrochemical detection of various compounds related to biological processes (i.e., hydrogen peroxide [9,10], glucose [11], dopamine [12], etc.). In addition to this, magnetic nanocomposites applied in biological systems have also shown great potential for molecular diagnosis of human diseases [13] and the development of advanced ultrasensitive techniques [14].

From a different point of view, it is known that bismuth (Bi) forms “fusible alloys” with heavy metals and exhibits good electrochemical and catalytic properties. Thus, Bi is considered one of the most efficient, low-toxic, and environmentally friendly compounds currently used for the electrochemical detection of heavy metals [15,16,17].

During previous studies, the sol-gel synthesis was modified in order to add the benefits of Fe and Bi nanoparticles to the carbon xerogel matrix (CXBiFe) [18,19]. Variations in the nanocomposite structure, morphology, and composition induced by chemical synthesis [18,19] and drying steps [16] were analyzed and correlated with the electrochemical response of nanocomposite-modified electrodes. The pyrolysis-induced effects are in terms of phase changes and nanoparticle dynamics, i.e., related to hybrid Bi and Fe oxides (BFO) nanoparticles and Fe-induced graphitization, but without an electrochemical approach [20]. The nanocomposites were classified by considering a four-stage thermal evolution observed during in-situ transmission electron microscopy (TEM) experiments: (1) precursor decomposition (~100–250 °C), (2) carbonization (~250–750 °C), (3) reduction (~600–800 °C), and (4) Fe-assisted graphitization (~800–900 °C) [20].

In the present study, we further investigate the multi-scale effects by which the pyrolytic treatment of CXBiFe nanocomposites will influence the electrochemical behavior of glassy carbon electrodes modified with CXBiFe incorporated in a chitosan (Chi) matrix. Nanocomposites, denoted as CXBiFe-T, are pyrolized as xerogel monoliths under Ar purge at different temperatures (where T is 600, 750, 900, or 1050 °C). An improved electrochemical response is observed and correlated with nanocomposite multiscale changes obtained by using several techniques such as X-ray diffraction (XRD), Raman spectroscopy, N_2_ adsorption, dynamic light scattering (DLS), SEM/EDX, and TEM microscopies. Their use as sensors for amperometric detection of Pb^2+^ showed notable performances in terms of detection limit and sensitivity. At the same time, the presence of Fe nanoparticles in the sensing matrix of GC/Chi-(CXBiFe) electrodes allowed H_2_O_2_ amperometric detection, which supports the potential usage of CXBiFe-modified electrodes as multifunctional sensors.

## 2. Results and Discussion

### 2.1. Morphological and Structural Characterization

#### 2.1.1. Pyrolysis-Induced Changes in the Crystalline Structure

**Figure 1:**
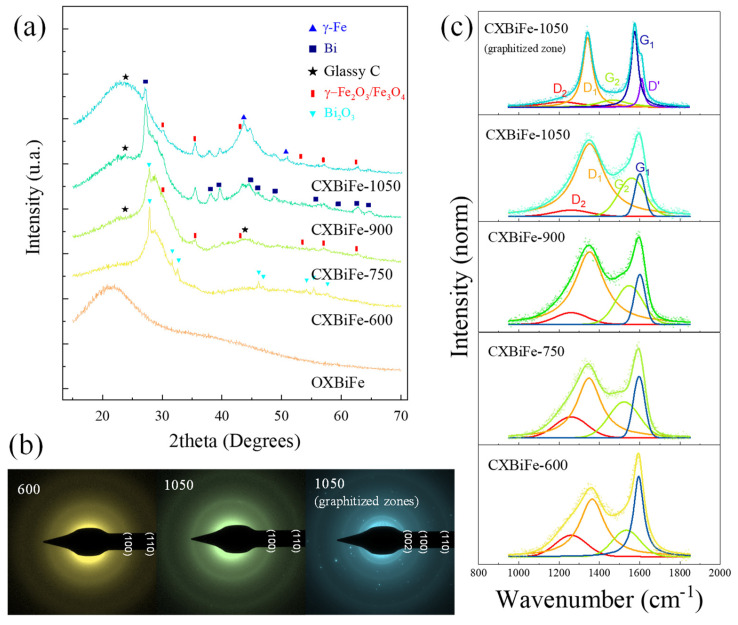
Pyrolysis-induced effects over the crystallinity and structural features of CXBiFe−T nanocomposites were observed in (**a**) XRD profiles compared with the reference patterns for β−Bi_2_O_3_, Bi, γ−Fe_2_O_3_/Fe_3_O_4_, and γ-Fe; (**b**) SAED patterns obtained for the CXBiFe−600 and CXBiFe−1050 samples; and (**c**) Main carbon signals from the Raman investigations of CXBiFe−T samples and deconvolution results.

The XRD patterns are shown in Figure 1a. The diffractogram of the as-prepared organic xerogel nanocomposite (OXBiFe) reveals only a broad amorphous hallow centered at 2θ ≈ 21.60° (d_RF_ ≈ 4.11 Å). 

After the pyrolysis at 600 °C, the meta-stable tetragonal β-Bi_2_O_3_ phase is dominant (strongest reflection located at 2θ = 27.8°), with an additional amorphous hallow around 2θ = 28.7°. After the 900 °C pyrolysis, changes indicating the Bi_2_O_3_ reduction to metallic Bi are finally observed. The signal found at 2θ = 35.4° represents the scattering from the (311) planes in iron oxide, while the small reflections observed at 2θ = 43.69° and 2θ = 50.80° trace to the (111) and (200) planes, specific to γ-Fe. At 1050 °C, the amorphous oxide signal is completely lost, the intensity of the Bi reflections also decreases, and the iron phases are refined. The average crystallite size (d_XRD_) for Bi and Fe-based crystalline phases was determined by using the well-known Scherrer equation (see Table 1). The data indicate that, after reduction processes, a size decrease is observed both for Bi and Fe phases, consistent with the previous findings [20]. 

Considering the carbon support, during pyrolysis of RF xerogels, the organic components thermally degrade and form a carbonaceous network of basic structural units (BSU) based on stacked honeycomb structures with large defect concentrations and an average stacking distance of d_002_ ≈ 3.8 Å. The selected area electron diffraction (SAED) patterns trace an evolution of the carbon support from predominantly amorphous features at 600 °C to well-defined (100) and (110) reflections due to higher nanocrystalline order at elevated temperatures (Figure 1b) [21]. For some regions found in the CXBiFe-1050 sample, the SAED patterns also contain a high-intensity (002) diffraction ring associated with stacked graphitic planes. 

The first-order region of the Raman spectra showing the carbon-specific D-G bands is presented in Figure 1c. After a 4-band deconvolution described in the Supplementary Section (Table S1), parameters such as the intensity ratio between the D_1_ and G_1_ bands (I_D1_/I_G1_) and the G_1_ position can be used to evaluate the level of disorder. The I_D1_/I_G1_ ratio (see Table 1) is observed to increase with pyrolysis temperature from 0.72 for CXBiFe-600 to 1.70 for CXBiFe-1050, along with blue-shifts in peak position (1595 to 1601 cm^−1^), while the FWHM values were found to decrease. Based on the valuable Raman investigations performed by Ferrari et al., these trends are specific to sp^2^-rich nano-crystalline carbons that show an increase in the I_D1_/I_G1_ ratio with the crystallinity level [22]. This is also supported by the previously described SAED results. Further on, some regions observed in the CXBiFe-1050 sample exhibited even higher graphitization yields due to the catalytic role of Fe nanoparticles (Figure 1c—top).

#### 2.1.2. Effects Induced at Micro- and Nano-Scales

**Figure 2:**
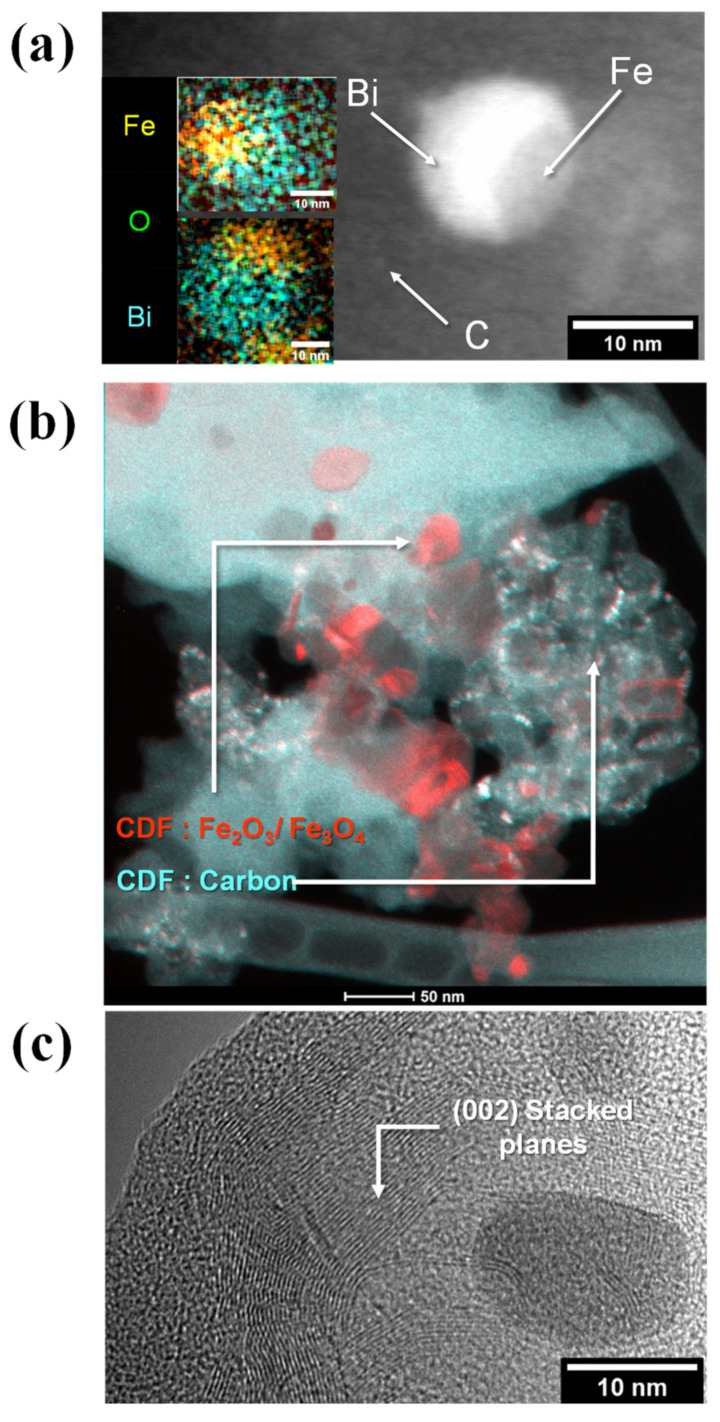
TEM investigations of CXBiFe-T nanocomposites show (**a**) BFO nanoparticles and basic structural units of the carbon support found in CXBiFe-T samples as imaged in HAADF-STEM mode. The insets represent EDX spectral images depicting the distribution of Bi (cyan), O (green), and Fe (orange) obtained for CXBiFe-750 and CXBiFe-900, respectively; (**b**) Superposed conical dark field micrographs of graphitized zones with carbon nanostructures (cyan) and iron-based nanoparticles (red) in CXBiFe-1050; and (**c**) HRTEM images at the interface between the turbostratic carbon nanostructures (002 stacks) and the glassy carbon support.

During TEM and STEM investigations, a dumbbell/spheroid morphology was observed, especially for nanoparticles found at lower pyrolysis treatments (Figure 2a). The confirmed presence of BFO nanoparticles may explain the amorphization of the Bi phases at lower temperatures, as discussed in earlier studies [18,20]. As higher reduction yields are obtained at greater temperatures, a separation between the Fe-rich and Bi-rich phases is expected [18].

Under STEM imaging, the BSUs constituting the nano-crystalline carbon support were also resolved with a size smaller than 3 nm, as shown in Figure 2a. No clear temperature-induced variations in BSU size could be emphasized, but graphitized carbon nanostructures were observed for the CXBiFe-1050 sample using conical dark field imaging and HRTEM, as presented in Figure 2b,c. The occurrence of graphitic nanostructures is a sign of enhanced iron oxide reduction and activation of the graphitization mechanism, which may lead to enhanced electric properties.

The specific surface area (S_BET_) values for the treated samples were found to be high (Table 1), due to activation and thermal degradation of the organic matrix, while a notable decrease is observed with the pyrolysis temperature, which was interpreted as a nano-scale densification of the carbon matrix [7,23] due to structural reorganizations of the carbon network and micropore collapse [7,23]. 

**Figure 3:**
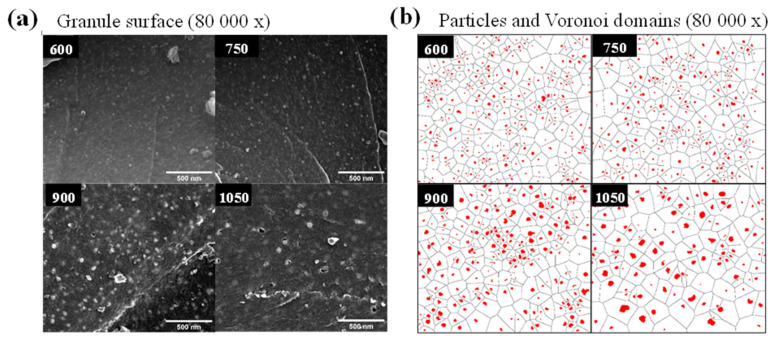
CXBiFe−T samples at microscopic and nanoscales during SEM analysis (**a**) surface of CXBiFe-T grains at 80,000× evidencing the distribution of nanoparticles (**b**) results of the image processing sequence for particle detection and Voronoi tessellation for the same micrographs.

The pyrolysis-induced changes that occur at the micro- and nano-metric scales were investigated in detail using SEM investigations on CXBiFe-T monoliths before and after grinding (Figure S1), while the ground nanocomposite aggregate size distribution was obtained from DLS measurements (Figure S2). The DLS results mainly indicate a decrease in the mean aggregate size from ~1.40 µm to 0.57 µm. The SEM-EDX investigations at the cross-section of CXBiFe-T monoliths (having a sampling range of 1–3 mm, as seen in Figure S1a) were further used to classify the components from the nanocomposite xerogel into (1) carbonaceous granules with embedded metal/oxide nanoparticles, (2) scattered microparticles from the external crust, and (3) Bi micro-spikes (Figures S3–S5). The carbonaceous granules represented by far the dominant feature of the nanocomposite monolith and were further investigated using SEM at higher magnifications (Figure 3a). For nanoparticle segmentation, size distribution analysis, and Voronoi tessellation [24], the micrographs were analyzed using the image processing sequence (see Figure S6 and the explanatory section from SI). The results are shown in Figure 3b. One can observe a trend of decrease with temperature in the nanoparticle population densities from roughly 80 np/µm^2^ (for CXBiFe-600) to 40 np/µm^2^ (for CXBiFe-1050). This is obviously associated with the temperature-dependent growth, diffusion, and coalescence processes [20]. The mentioned processes also impact nanoparticle size and inter-particle distancing, as the nanoparticle size histograms (PSH) and Voronoi domain size histograms (VDSH) shown in Figure S7 are widened and shift towards larger sizes with temperature. The average nanoparticle size (d_np_) and the average Voronoi domain size (D_V_) are summarized for each CXBiFe-T nanocomposite in Table 1. Corroborated with our earlier studies [20], it is expected that the microscopic coverage of the accessible carbon surface with metal/oxide nanoparticles (θ) will exhibit a maximum at intermediate temperatures somewhere in the given range of temperatures, followed by a decrease due to reduction and Bi losses. As later presented, the microscopic coverage and the PSH and VDSH parameters are considered crucial in describing the microscopic coverage of electrochemically active metal/oxide centers during voltammetry experiments at heterogeneous electrodes [25]. 

### 2.2. Electrochemical Behavior of GC/Chi-(CXBiFe-T) Modified Electrodes

#### 2.2.1. EIS Measurements

**Figure 4:**
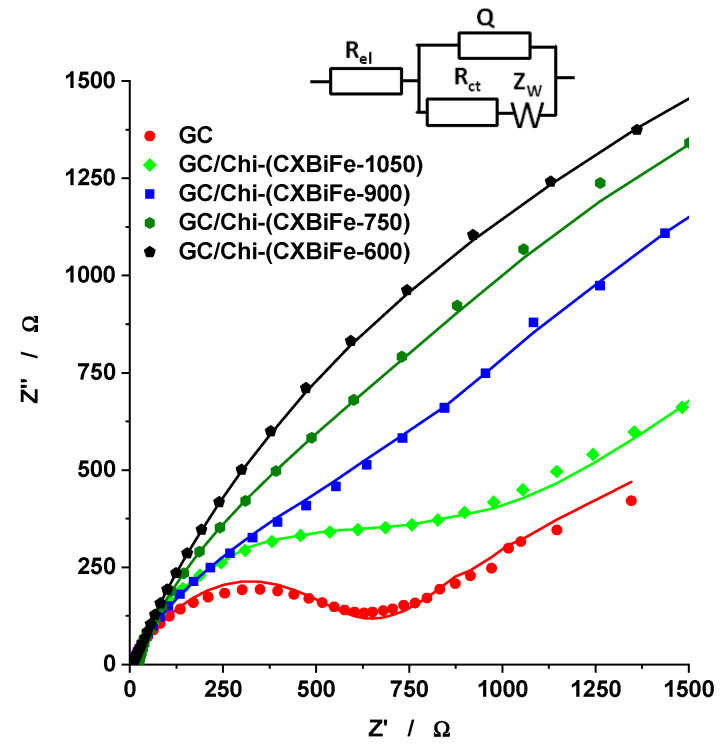
Impedance spectra recorded at GC/Chi-(CXBiFe-T) modified electrodes. Experimental conditions: supporting electrolyte, 0.1 M acetate buffer (pH 4.5) containing 5 mM [Fe(CN)_6_]^3−/4−^; applied potential, 0.215 V vs. Ag/AgCl, KCl_sat_; frequency interval, 0.1–10^4^ Hz.

The electrochemical response of the GC/Chi-(CXBiFe-T) modified electrodes was examined using EIS measurements [4,5,26], performed in the presence of [Fe(CN)6]^3−/4−^ (Figure 4). The results were best fitted to a modified Randless equivalent circuit involving an uncompensated electrolyte solution resistance (R_el_) coupled in series with the parallel arrangement of the mixed capacitance (containing the constant phase element (CPE) and the double layer capacitance (C)) and the faradaic resistance (consisting of the charge transfer (R_ct_) and the mass transfer (W) resistances) (Figure 4). Table 2 summarizes the estimated values for all the above-mentioned electrochemical parameters. 

The increase in pyrolysis temperature of the CXBiFe nanocomposites induces an exponential decrease in R_ct_ values, associated with somewhat lower values of double layer capacitance. To the best of our knowledge, such dependence was reported for the first time for carbon-based nanocomposite. One possible explanation is that pyrolysis at higher temperatures produces important structural changes that govern the electron transport properties of the material, such as the transition from a non-graphitic disordered structure with atomic-scale randomness to nano-crystalline graphite domains [27]. 

#### 2.2.2. Square Wave Voltammetry Measurements for H_2_O_2_ Detection

Considering the presence of Fe nanoparticles in the CXBiFe nanocomposites structure and based on the results obtained from the analysis of the EIS measurements, the electrode with lower R_ct_ and Q values, i.e., the GC/Chi-(CXBiFe-1050) modified electrode, was selected for H_2_O_2_ detection by using the SWV method. The SW voltammograms recorded at the GC/Chi-(CXBiFe-1050) electrode in H_2_O_2_ presence show well-shaped cathodic peaks strongly correlated with the H_2_O_2_ concentration (Figure S8a). As expected, these peaks are placed in the potential domain corresponding to the voltammetric signal of Fe oxides present in the CXBiFe nanocomposite matrix (see the SW voltammogram recorded in the absence of H_2_O_2_). The calibration curve (Figure S8b) was obtained by using the average of the peak current intensities recorded for three different GC/Chi-(CXBiFe-1050) electrodes.

The analytical parameters estimated for H_2_O_2_ detection at the GC/Chi-(CXBiFe-T) investigated electrodes are summarized in Table 3. The GC/Chi-(CXBiFe-1050) electrode provided the best results in terms of sensitivity and limit of detection, confirming the beneficial effect of the high pyrolysis temperature of CXBiFe nanocomposites in the case of H_2_O_2_ detection. Compared with the results reported for similar electrodes (Table 3), those obtained for GC/Chi-(CXBiFe-1050) modified electrodes are characterized by a relatively low detection limit, good sensitivity, and a relatively extended linear domain.

#### 2.2.3. Square Wave Anodic Stripping Voltammetry Measurements for Pb^2+^ Detection

**Figure 5:**
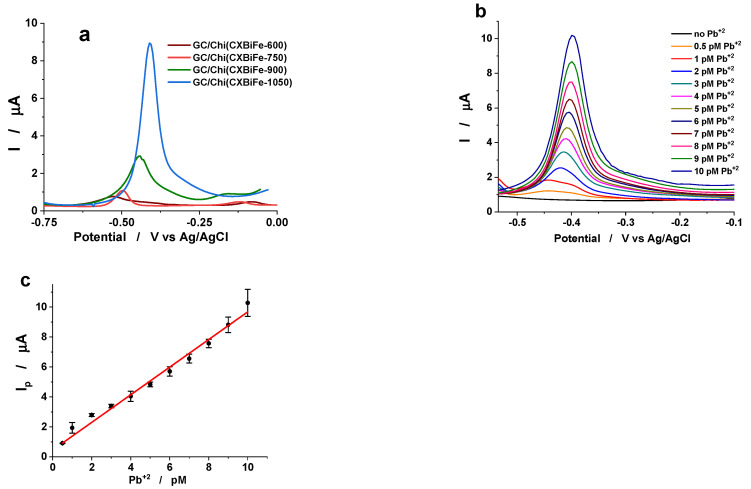
(**a**) SWASV recorded at GC/Chi-(CXBiFe-T) modified electrodes in the presence of 10 pM Pb^2+^; (**b**) SWASV recorded at GC/Chi-(CXBiFe-1050) electrodes for different concentrations of Pb^2+^; (**c**) The corresponding calibration curve for Pb^2+^. Experimental conditions: supporting electrolyte, 0.1 M acetate buffer (pH 4.5); deposition potential, −1.4 V vs. Ag/AgCl, KCl_sat_; deposition time, 180 s; frequency, 10 Hz; amplitude, 25 mV.

Well-shaped anodic peaks, corresponding to the Pb dissolution previously deposited on the electrode surface during the preconcentration step, were observed for all GC/Chi-(CXBiFe-T) modified electrodes. However, as can be seen from Figure 5a, the best SWASV results were recorded at GC/Chi-(CXBiFe-1050) modified electrodes. Thus, for the same Pb^2+^ concentration (10 pM), the peak current corresponding to the Pb dissolution at GC/Chi-(CXBiFe-1050) was 11.2 times higher than that recorded at GC/Chi-(CXBiFe-600).

The outstanding behavior of GC/Chi-(CXBiFe-1050) modified electrodes for Pb^2+^ detection at very low levels of concentration (0.5–10 pM) is illustrated in Figure 5b. The average results obtained by performing SWASV measurements at all GC/Chi-(CXBiFe-T) modified electrodes were used to draw the linear calibration curve, expressed by the parameters presented in Table S2. For three different GC/Chi-(CXBiFe-1050) electrodes, the calibration curve depicted in Figure 5c leads to the electroanalytical parameters for Pb^2+^ detection (presented in Table 4).

**Figure 6:**
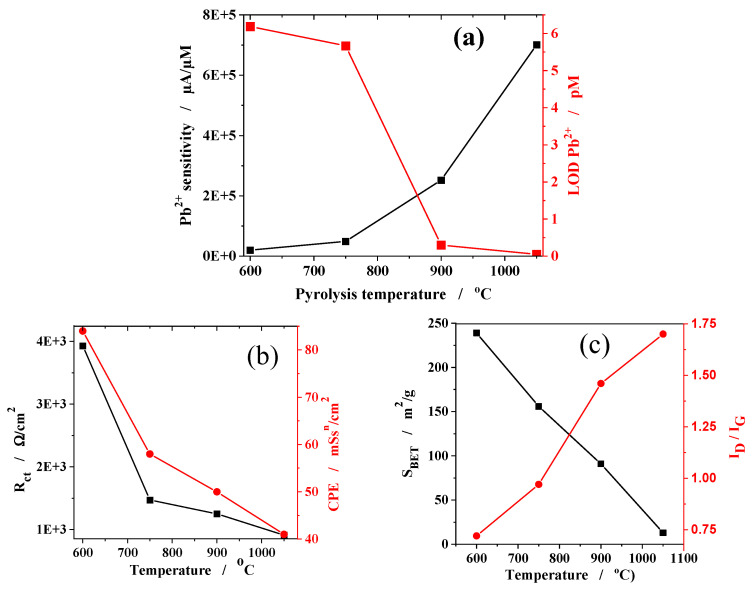

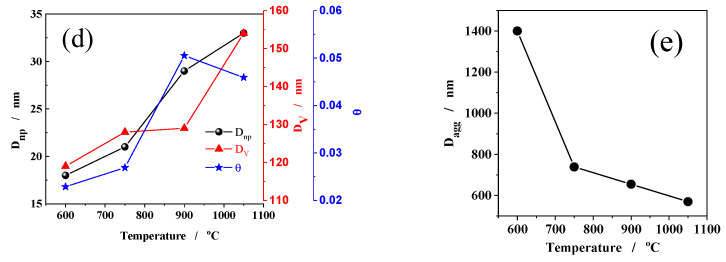
Temperature dependence for different morpho-structural and electrochemical parameters: (**a**) Sensitivity_Pb2+_ and LOD_Pb2+_ from SWASV; (**b**) R_CT_ and CPE from EIS; (**c**) S_BET_ from N_2_ adsorption and the I_D_/I_G_ ratio from Raman fit results; (**d**) average nanoparticle size (D_np_), Voronoi domain size (D_V_), and microscopic coverage (θ) from SEM; and (**e**) average CBiFe aggregate size (D_agg_) from DLS. The standard deviations for the values represented in (**a**–**c**) do not exceed ±4%.

It is noteworthy to mention that the increase in pyrolysis temperature has a positive effect on the Pb^2+^ detection sensitivity (Figure 6a). Thus, the sensitivity estimated for the GC/Chi-(CXBiFe-1050) electrode was 46 times higher than the sensitivity measured for the GC/Chi-(CXBiFe-600) electrode. At the same time, the detection limit for the GC/Chi-(CXBiFe-1050) electrode (97 fM, see Table 4) was found to be 63 times lower than that achieved for the GC/Chi-(CXBiFe-600) electrode (6.19 pM, see Table 4) (both estimated for a signal-to-noise ratio of 3), and the obtained values are presented in Figure 6a. Comparing the main analytical parameters, estimated for all GC/Chi-(CXBiFe-T) modified electrodes (Table 4), it is interesting to notice: 

(i) The increase in pyrolysis temperature is associated with a positive shift in the peak potentials for Pb dissolution deposited from Pb^2+^ solutions in contact with GC/Chi-(CXBiFe-T) electrodes (Figure 5a and Table 4). Among other effects, the heat treatment of the carbon-based materials under an argon atmosphere reduces the number of superficial groups containing oxygen [32,35] and increases the surface hydrophobicity [14]. The increase in surface hydrophobicity with the pyrolysis temperature may be responsible for the positive shift of the peak potential.

(ii) To the best of our knowledge, the Pb^2+^ detection limit estimated for the GC/Chi-(CXBiFe-1050) modified electrode has the lowest ever reported value (97 fM). Moreover, the detection limit found for the GC/Chi-(CXBiFe-900) modified electrode (0.3 pM) is well placed among the best reported results (Table 4).

(iii) The significant difference between the sensitivities observed for Pb^2+^ detection at GC/Chi-(CXBiFe-600) or GC/Chi-(CXBiFe-750) and GC/Chi-(CXBiFe-900) or GC/Chi-(CXBiFe-1050) (Table 4) emphasizes once again the crucial role of the thermal treatment of the CXBiFe nanocomposites.

#### 2.2.4. Correlations between the Morphological and Structural Characteristics and the Electroanalytical Behavior of BiFe-Carbon Xerogel Nanocomposites Modified Electrodes

Possible correlations between the electroanalytical parameters for Pb^2+^ detection and a broad set of morphological, structural, and electrochemical parameters that are dependent on pyrolysis temperature are presented in Figure 6. 

**Figure 7:**
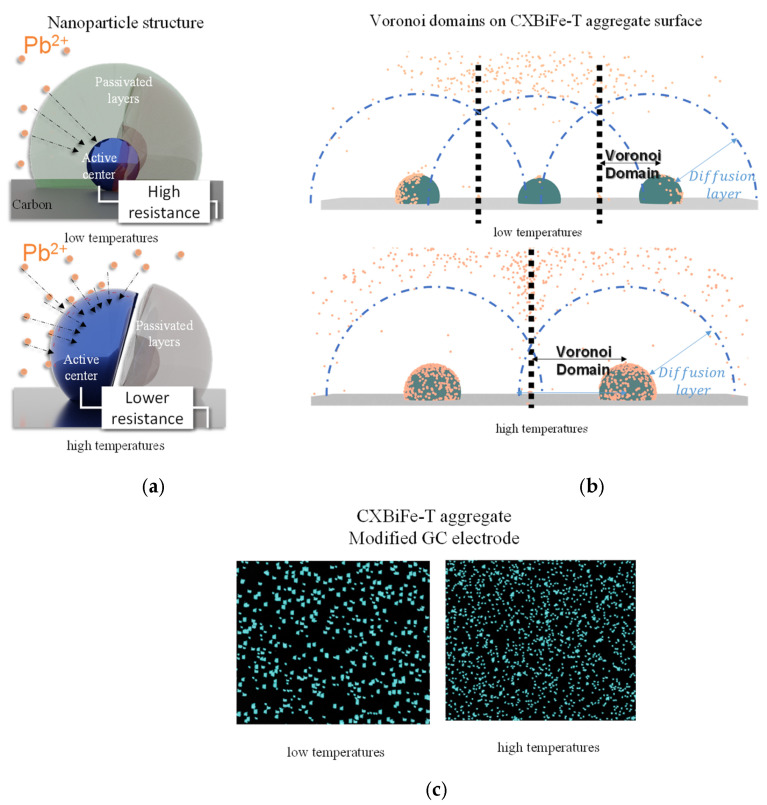
Models for pyrolysis effects taking place at different spatial scales for low and high temperatures in CXBiFe-T nanocomposite systems: (**a**) compactization, graphitization, and nanoparticle activation; (**b**) spatial statistics and diffusion domains; and (**c**) micro-scale granularity changes for CXBiFe-T aggregates.

Since the pyrolysis treatment will influence the nanocomposite systems at different scales, the findings are further discussed from a bottom-up perspective, as illustrated in Figure 7.

##### Conductivity and Porosity

The conductive properties of the carbon support are strongly linked with the data derived from EIS spectroscopy (Figure 6b), Raman spectroscopy, and N_2_ adsorption (Figure 6c). The R_CT_ and CPE decrease non-linearly, while the Raman-derived I_D1_/I_G1_ ratio and the S_BET_ values behave almost linearly with the pyrolysis temperature. The results suggest that the electric connectivity between the metal/oxide centers and the carbonaceous support is enhanced during pyrolysis by various means, such as the insulator-to-conductor transition [36,37], increased levels of graphitization, and densification effects [7]. Such effects were traced towards higher sensitivities in other systems as well [38]. Even though improved charge transport efficiency may have a major contribution to the variation of sensor response with temperature, the results from Figure 6b,c explain only to some extent the trends observed in the electrochemical Pb^2+^ detection (Figure 6a).

##### Nanoparticle Activation and Spatial Statistics of Diffusion Domains

At the nanoscale, it was found from XRD and electron microscopy analyses that pyrolysis affects the structure of the nanoparticles in terms of their level of reduction and purity (Figure 7a). In the composites treated at lower pyrolysis temperatures (CXBiFe-600 and CXBiFe-750), bismuth was mostly found in amorphous and crystalline oxide states and in hybrid BFO nanoparticles. When investigating the effect of iron content on composites obtained at 750 °C, we observed that H_2_O_2_ detection improved with iron concentration, while an opposite trend was obtained for Pb^2+^ detection [18]. 

In the present study, even though higher Bi concentrations were found in the cases of CXBiFe-600 and CXBiFe-750, the interactions in BFOs may reduce the electrochemically active surface of individual Bi nanoparticles. At higher temperatures, during the reduction stage, i.e., for CXBiFe-900 and CXBiFe-1050, when the melting temperatures of Bi_2_O_3_ [39] or the peritectic decomposition of Bi_2_Fe_4_O_9_ [40] are reached (T_Bi2O3_ ~ 824 °C and T_BFO_ ~ 825–937 °C, respectively), a separation of the hybrid BFO nanoparticles into Bi rich and Fe rich nanoparticles is expected to take place, as observed elsewhere [20], thus leading to more available electroactive centers and improved performances in Pb detection (illustrated in Figure 7a). As discussed above, this effect is not fully quantified and should be further investigated in future studies.

Another important aspect concerns the temperature variation of the SEM-derived parameters regarding average nanoparticle size (d_np_), Voronoi domain size (D_V_), and the microscopic surface coverage parameter (θ), as illustrated in Figure 7b. The trends indicate a non-linear increase in microscopic surface coverage θ that also correlates well with the trends in electrochemical detection (see Figure 6a,d). Considering the previously presented SEM analysis, the growth pattern specific to the metal centers is associated with enhanced migration and coalescence of molten fractions. This will lead to different levels of occupancies for the Voronoi domains for each type of CXBiFe-T composite and may ultimately lead to improved Pb^2+^ harvesting due to the smaller overlap of diffusion domains (Figure 7b). If Bi losses due to melting and coalescence persist at a higher level, i.e., applying longer treatments at elevated temperatures, the Pb detection yields are expected to drop. 

A last significant contribution is brought about by the pyrolysis effects on the nanocomposite grains at microscopic scales. As observed during the SEM and DLS analyses (Figure 6e), grinding the samples obtained at higher temperatures will induce smaller nanocomposite grain sizes. During electrode assembly, constant volumes of Chit-CXBiFe-T solutions (obtained by keeping the same CXBiFe-T mass for all systems) are drop-casted onto the glassy carbon surface. For systems such as GC/Chi-CXBiFe-900 or GC/Chi-CXBiFe-1050, this will lead to a higher number of fine granules deposited onto the glassy carbon electrodes, expose more Bi sights, and increase the geometric areas of the electrode, as illustrated in Figure 7c. 

It is thus necessary to remark that the high performances obtained for such systems rely on a complex architecture characterized by parameters defined at multiple scales, notably all necessary in describing the detection efficiency, all influenced by pyrolysis and processing conditions. To fully support these claims, more in-depth investigations are required. i.e., by applying a theoretical model based on random arrays of Bi and/or Fe-based nanoelectrodes that should consider both the aforementioned parameters as well as the diffusion layer thickness, the local surface coverage, and the macroscopic coverage that characterize such experiments [25,41,42]. 

#### 2.2.5. Sensor—Long-Term Stability and Repeatability

The sensor’s long-term stability was examined immediately and after five months since sensor preparation by recording the SWASV responses at the GC/Chi-(CXBiFe-1050) in contact with a 9 pM Pb^2+^ standard solution (the experimental conditions are specified in Figure 5). The average of peak currents, resulting from three different measurements, showed a slight relative decrease of ~3.5%. 

The sensor repeatability was investigated for the GC/Chi-(CXBiFe-1050) electrode by performing successive measurements in the presence of a 9 pM Pb^2+^ standard solution (under the same experimental conditions as those mentioned in Figure 5). The relative standard deviation between four consecutive measurements was lower than ~1.2% (Table S3). 

Taking into consideration the long-term stability and repeatability of this sensor, the design of the next-generation of conductor surfaces or tissue engineering materials can become a reality. In this respect, the analysis of different mixtures of polymer electrolytes [43] and hydrocolloids [44] could contribute to the development of such future materials. 

#### 2.2.6. Real Sample Analysis

In order to prove the applicability of the GC/Chi-(CXBiFe-1050) modified electrode for Pb^2+^ detection, two samples of drilled well water (Jibou, District of Salaj, Romania) were used. By using the standard addition method, the Pb^2+^ concentration was estimated. The SWASV recordings were performed under similar experimental conditions as for the electrode calibration against Pb^2+^. The obtained results were in very good agreement with those obtained by the standardized technique used for drinking water investigation by the provider of water samples (atomic absorption spectroscopy) (Table S4).

## 3. Conclusions

The present study outlined the electrochemical sensing performances of carbon xerogels with bismuth and iron-based nanoparticles obtained under different thermal stages of evolution during pyrolysis. By using SWV for two different analytes (Pb^2+^ and H_2_O_2_) detection at glassy carbon electrodes modified with chitosan and CXBiFe nanocomposites, it was proven that the pyrolysis temperature of CXBiFe exerts a crucial influence on the analytical parameters of the resulting sensors. The exponential increase in Pb^2+^ sensitivity with temperature was explained using several multi-scale effects, including (1) the transition from the amorphous carbon network to densified, nano-crystalline graphites; (2) the reduction of BFO hybrid nanoparticles, the splitting and activation of new Bi and Fe-rich sites; and (3) changes in the spatial distribution of electrochemically active centers and in aggregate size. A higher thermal treatment leads to nanocomposites found in an advanced reduction/incipient graphitization stage and improved electrochemical response. 

The preparation procedure of the investigated electrode material avoids the presence of noble metals, enzymes, or other carbon-based nanomaterials (CNT, graphene, etc.), and exploiting a one-step sol-gel synthesis associated with the use of very small quantities of electrode material leads to the obtaining of very competitive sensors for two important and representative analytes, Pb^2+^ and H_2_O_2_. 

To the best of our knowledge, the cumulative effect of the thermal treatment temperature over the particularities of electrode materials and the detection of two analytes belonging to two important groups—heavy metals (Pb^2+^) and biomarkers (hydrogen peroxide)—were analyzed here for the first time.

At the same time, it is worth mentioning that the lowest detection limit for Pb^2+^ detection (97 fM) was reported, while for H_2_O_2,_ a relative low detection limit (2.51 µM) was found associated with a linear range (50–1000 µM), well placed in the domain of practical interest for biochemical studies.

## 4. Materials and Methods

### 4.1. Reagents

Reagents were used without any further purification: resorcinol (99%, Sigma-Aldrich, Saint Louis, MO, USA), formaldehyde solution (37 wt% in H_2_O, Sigma-Aldrich, Saint Louis, MO, USA) stabilized with methanol (CH_3_OH, Chem-Lab NV, Zedelgem, Belgium); CH_3_COOH (99.7%), Fe(OOCCH_3_)_2_ (minimum Fe content 29.5%), (CH_3_COOH, 99%), NH_4_OH (10 wt%), 47–67% 5-hydroxy-1,3-dioxane, 33–53% 4-hydroxymethyl-1,3-dioxolane and Bi(NO_3_)_3_·5H_2_O, 98% were purchased from Alfa Aesar GmbH and Co KG, Haverhill, MA, USA. All reagents were of analytical grade. Bidistilled water was used for the preparation of all solutions.

### 4.2. Synthesis and Temperature Program for CXBiFe-T Xerogel Ternary Composite

Resorcinol (2 g, 18.17 mmol) was added to a preformed solution of Bi(NO_3_)_3_·5H_2_O (1.20 g, 2.47 mmol) dissolved in glycerol formal (10 mL) under vigorous stirring at room temperature. To this orange solution, formaldehyde (2.72 mL, 36.52 mmol) was further added. During the polycondensation of resorcinol with formaldehyde, bismuth salt plays both the role of acidic pre-catalyst and bismuth source, while glycerol formal is the reaction solvent. To obtain a clear, transparent solution through a pH adjustment, ammonium hydroxide (10%, 4 mL) and glacial acetic acid (12 mL) were consecutively added. Then, the addition of iron acetate (1.20 g, 6.89 mmol) was performed. To reach the gelation point, the solution was stirred for ten minutes and then cured in a sealed glass cylinder at 60 °C for 72 h. The obtained Bi^3+^ and Fe^2+^ modified organic gel was washed several times in acetic acid and ethanol. 

After drying in the air at room temperature for 3 days, the obtained monolithic xerogel was pyrolyzed in an Ar atmosphere at various temperatures: 600, 750, 900, and 1050 °C. The temperature program consisted of a 2 h platform at 250 °C, followed by 3 °C/min heating to reach the final temperature, where the samples swelled for 2 h. The resulting CXBiFe-T samples (where T = 600, 750, 900, and 1050 °C) preserved their monolithic structure, as presented in Figure S9. Prior to the physical or electrochemical investigations, the samples were ground with a Retsch MM 200 Mixer Mill for 15 min at an oscillation frequency of 20 Hz to produce a finely granulated and homogenized powder. 

### 4.3. Characterization Methods

X-ray diffraction (XRD) measurements were performed with a Shimadzu 6000 diffractometer (Kyoto, Japan) using Cu-Kα radiation (λ = 1.5406 Å) equipped with a graphite monochromator.

Raman spectra were measured with a Renishaw Via Reflex Raman Microscope. As an excitation source, a 532 nm laser was used at a power of 1 mW. The spectra were collected using a 0.85 NA objective at 100× magnification and a Ren Cam CCD detector, considering integration times of 30 s. The spectral resolution was 4 cm^−1^. The spectra were fitted according to a fit procedure described in other works [45].

The granule size distributions were measured at room temperature by using a Nano-ZS90 model of the Malvern Zetasizer Nano instrument after the samples were dispersed in an aqueous medium at a concentration of 1 mg/mL. 

The specific surface area of the sample was determined by measuring nitrogen adsorption/desorption at 77 K with the surface area analyzer Qsurf Series M1, on the basis of the Brunauer, Emmet, and Teller (BET) equation, S_t_ = K (1 − P/P_o_)V_a_, with S representing the total surface area; K = 4.35, the constant specific for nitrogen, while assuming the STP condition; P/P_o_—the relative pressure (0.294) for a 30% N_2_/70% He gas mixture; and V_a_—the volume of gas (N_2_) adsorbed. The measurements relied on the one-point BET method, considering a gas composition of 30% N_2_ and 70% He. 

A FEI Quanta 3D FEG dual beam scanning electron microscope was operated in high vacuum mode using the EDT (Everhart Thornley Detector) and ApolloX SDD Energy Dispersive X-ray (EDX) detectors. To investigate the thermal effects induced by the spatial distribution and particle size, the micrographs of flat grain regions were analyzed using FIJI 1.53 software [46,47]. Briefly, the image processing sequence detailed in the Supplementary Section (see Figure S6) was used to detect populations between 150 and 250 nanoparticles with a size range between 10 and 70 nm. The images were binarized in order to determine the size, position, and Voronoi domain of each nanoparticle, later representing the locus of points found nearest to one particle than to any other. The average microscopic coverage of metal/oxide centers (θ) is further defined as $\theta ={\left(\frac{d_{np}}{D_{V}}\right)}^{2}$, where d_np_ and D_V_ represent the averages of nanoparticle size and Voronoi domain size, as considered elsewhere [48].

The transmission electron microscopy (TEM) micrographs were acquired in bright field (BF), conical dark field (CDF), and high resolution (HRTEM) modes, together with selective area electron diffraction patterns (SAED), using FEI Tecnai G2 F20 X-Twin TEM operating at 200 kV. The Z-contrast micrographs and EDX chemical maps were obtained using a Hitachi HD-2700 CFEG Scanning Transmission Electron Microscope (STEM) equipped with two 100 mm^2^ windowless SD detectors from Oxford Instruments operating at 200 kV. 

### 4.4. Preparation of the GC/Chi-(CXBiFe-T) Electrodes

Before each measurement, the glassy carbon electrode (GCE, with a geometrical area of 0.07 cm^2^) was carefully polished with alumina slurry (1 μm and then 0.1 μm, Stuers, Copenhagen, Denmark). Afterwards, the GCE was washed with bidistilled water and sonicated for 5 min in acetone in order to remove alumina particles and other possible contaminants. To immobilize the nanocomposites onto the GCE surface, each of the CXBiFe-T nanocomposites was dispersed in polymer solutions consisting of 10 mg chitosan (Chi) and 10 mL of acetic acid (0.1 M). The mixture resulted from adding 1 g/L CXBiFe-T and was sonicated for 2 h. The obtained suspensions were placed on the cleansed GCE surfaces and left to dry under a beaker at room temperature. 

### 4.5. Electrochemical Measurements

The electrochemical measurements were performed by an electrochemical analyzer (AUTOLAB PGSTAT302N EcoChemie, Utrecht, The Netherlands). A three-electrode cell, having as a working electrode each of the modified electrodes (GC/Chi-(CXBiFe-T)), as a counter electrode, a Pt plate, and as a reference electrode, Ag/AgCl, KCl_sat_, was used. The EIS investigations were performed in a frequency range of 10^4^–10^−1^ Hz in acetate buffer (0.1 M, pH 4.5), having 5 mM [Fe(CN)_6_]^3−/4−^, in ambient conditions. The electroanalytical experiments were performed using different procedures, depending on the analyte solution (heavy metals or hydrogen peroxide). Thus, in order to detect heavy metal (i.e., Pb^2+^), the SWASV experiments were realized in acetate buffer (0.1 M, pH 4.5) after 180 s of potentiostatic polarization at −1.4 V vs. Ag/AgCl, KCl_sat_. The preconcentration step was performed under continuous stirring at 400 rpm. Then, 10 s before performing the anodic voltammetric scan, the stirring was stopped. H_2_O_2_ detection by SWV was realized in phosphate buffer (0.1 M, pH 7). The electroanalytical performance of GC/Chi-(CXBiFe-T) modified electrodes was confirmed for Pb^2+^ detection in the concentration range of 1–10 pM or 10–100 pM and 0.05–1 mM for H_2_O_2_, respectively. 

All experiments were carried out at the ambient temperature.

## Figures and Tables

**Table 1:** Morphological and structural parameters of CXBiFe-T nanocomposites.

Sample	d_XRD_ (nm)				I_D1_/I_G1_	S_BET_ (m^2^/g)	D_DLS_ (nm)	C/O/Bi/Fe (at%)	d_np_ (nm)	D_V_ (nm)
	Bi_2_O_3_	Bi	Fe_2_O_3_/Fe_3_O_4_	γ-Fe						
OXBiFe	-	-	-	-	0.86	10	-	74.0/25.5/0.2/0.2	-	-
CXBiFe-600	33	-	-	-	0.72	229	1400	93.6/5.6/0.4/0.5	18	119
CXBiFe-750	45	-	14	-	0.97	146	740	95.2/3.8/0.5/0.5	21	128
CXBiFe-900	-	18	13	13	1.46	91	655	92.3/7.1/0.3/0.3	29	129
CXBiFe-1050	-	22	18	13	1.70	16	570	94.4/4.8/0.1/0.7	33	154

d_XRD_, average crystallite size; I_D1_/I_G1_, ratio between D_1_ and G_1_ intensities; S_BET_, BET specific surface area; D_DLS_, average size of CXBiFe granules; C/O/Fe/Bi, EDX composition (in at%); C_BET_, d_np_, average nanoparticle diameter; and D_V_, average diameter of Voronoi domains.

**Table 2:** The parameters of the equivalent circuit (are shown in Figure 4).

Electrode Type	GC	GC/Chi-CXBiFe-600	GC/Chi-CXBiFe-750	GC/Chi-CXBiFe-900	GC/Chi-CXBiFe-1050
R_el_ (Ω cm^2^) *	5.98 ± 3	7.98 ± 0.9	22.6 ± 2.5	6.65 ± 1.5	12.2 ± 2.6
CPE (µS s^n^/cm^2^) * n *	25.2 ± 6 0.87 ± 1.0	84.1 ± 1 0.74 ± 0.22	58.2 ± 11 0.76 ± 2.1	50.2 ± 4 0.75 ± 0.79	40.9 ± 8 0.78 ± 1.4
R_ct_ (kΩ cm^2^) *	6.2 ± 2	3.93 ± 1.6	1.5 ± 15.6	1.25 ± 5.4	0.91 ± 4.3
W (mS s^1/2^/cm^2^) *	4.89 ± 5	0.38 ± 1	0.30 ± 6.7	0.76 ± 3.6	1.05 ± 3.7
C (µF/cm^2^)	13.3	56.6	26.4	19.5	16.5
χ^2^	0.003716	0.0002446	0.007471	0.002048	0.005647

* ±the relative standard deviations (%).

**Table 3:** Analytical parameters of amperometric sensors based on electrode nanomaterials incorporating Fe used for H_2_O_2_ detection.

Electrode Type	Peak Potential (V*)	Linear Range (mM)	Sensitivity (µA/mM)	Detection Limit (µM)	Ref.
(Fe-CA)-CPE	−0.3	1–50	1.78	500.0	[9]
α-Fe_2_O_3_NP|FePO_4_	−0.3	1.66–4.95	225.00	3.4	[28]
Fe_3_C/NG	−0.6	0.05–15	9.44	35.0	[29]
Fe-NGCs	−0.3	0.001–5	13.30	0.5	[30]
GC/Chi-BiFeCX	−0.3	0.005–0.05	1860.00	4.8	[19]
GC/Chi-CXBiFe1.2	−0.3	0.003–0.03	2350.00	0.2	[18]
GC/Chi-(CXBiFe-900)	−0.50	0.05–1	2.10	5.7	This work
GC/Chi-(CXBiFe-1050)	−0.46	0.05–1	4.55	2.5	

*V vs. Ag|AgCl, KCl_sat_; CPE, carbon paste electrode; NP, nanoparticles; Fe_3_C/NG, Fe_3_C-functionalized three-dimensional (3D) porous nitrogen-doped graphite carbon composites; Fe-NGCs, Fe-N-doped graphitic nanocages; GC, glassy carbon; GO, rGO, reduced graphene oxide; Chi, chitosan.

**Table 4:** Analytical parameters of amperometric sensors incorporating carbon-based nanomaterials proposed for Pb^2+^ detection.

Electrode Type	Peak Potential (V*)	Linear Range		Sensitivity (µA/µM)	Detection Limit (pM)	Ref.
		µM	pM			
GC/AlOOH-rGO	−0.6	0.2–0.8		3.5	93.20	[31]
Au/[Ru(bpy)_3_]^2+^-GO	−0.38	0.1–1.5		24.1	35.00	[32]
Fe_3_O_4_/Bi_2_O_3_/C_3_N_4_/GC	−0.5	0.01–3		82.5	10^3^	[33]
NiO/rGO/GCE		0.03–0.6		92.8	10^4^	[34]
GC/Chi-(BiCX)_Imp_	−0.55		1–10	1.1 × 0^6^	0.36	[15]
GC/Chi-(BiCX)_Cos_	−0.56		1–10	1.3 × 10^6^	0.28	[16]
GC/Chi-(BiCA)_Cos_	−0.44		1–10	2.3 × 10^5^	0.48	[16]
GC/Chi-(CXBiFe0.01)	−0.58		1–10	1.0 × 10^6^	0.56	[18]
GC/Chi-(CXBiFe-600)	−0.53		10–90	2 × 10^4^	6.19	This work
GC/Chi-(CXBiFe-750)	−0.48		10–110	4.9 × 10^4^	5.67	
GC/Chi-(CXBiFe-900)	−0.44		1–12	2.5 × 10^5^	0.30	
GC/Chi-(CXBiFe-1050)	−0.40		0.5–10	9.2 × 10^5^	0.09	

*V vs. Ag|AgCl, KCl_sat_; Imp, impregnation; Cos, cosynthesis; GC, glassy carbon; GO, graphene oxide; rGO, reduced graphene oxide; Chi, chitosan; [Ru(bpy)_3_]^2+^, Tris(bipyridine) ruthenium(II).

## Data Availability

Data are available upon request.

## Supplementary Materials

The following supporting information can be downloaded at: https://www.mdpi.com/article/10.3390/gels9110868/s1; Table S1. Four component fit results for the first order Raman spectra of CXBiFe at different pyrolysis conditions. The last set is characteristic to graphitized regions found on the surface of the monolith pyrolysed at 1050 C. Table S2. Linear regression parameters for SWASV detection of Pb^2+^ at CXBiFe-T nanocomposites modified glassy carbon electrodes. Table S3. Reproducibility of Pb^2+^ detection at GC/Chi-(CXBiFe-1050) modified electrodes. Table S4. SWASV recorded at GC/Chi-(CXBiFe-1050) and AAS results for Pb^2+^ determination in real samples of drilling water. Figure S1. CXBiFe-T samples at microscopic and nano-scales during SEM analysis (a) reconstructed wide range images of the CXBiFe-T monoliths, (b) SEM micrographs at 1200 x of CXBiFe-T powders after grinding. Figure S2. DLS measurements showing the size distributions of CXBiFe-T granules smaller than 5 μm. Figure S3. The main features observed for CXBiFe-T nanocomposites: carbon granules with embedded nanoparticles. Figure S4. Micron size spike formation at surface, cracks and micro-cavities in CXBiFe-T nanocomposites. Figure S5. Formation of oxidic crust towards the surface of CXBiFe-T nanocomposites. Figure S6. Image processing sequence for PSH and VDSH determination from SEM micrographs. Figure S7. PSH and VDSH obtained from SEM micrographs from the surface of CXBiFe-T granules. Figure S8. (a) SW voltammograms recorded at GC/Chi-(CXBiFe-1050) electrodes in presence of different concentrations of H_2_O_2_. Experimental conditions: supporting electrolyte, 0.1 M phosphate buffer (pH 7); frequency, 10 Hz; amplitude, 25 mV. (b) The corresponding calibration curve for H_2_O_2_ detection. Figure S9. Optical images of CXBiFe monoliths after pyrolysis at (a) 600, (b) 750, (c) 900, (d) 1050.
