# Supplementary material for: Ultrasensitive Electroanalytical Detection of Pb2+ and H2O2 Using Bi and Fe—Based Nanoparticles Embedded into Porous Carbon Xerogel—The Influence of Nanocomposite Pyrolysis Temperatures
Source: Gels. 2023 Oct 31;9(11):868. doi: 10.3390/gels9110868 (PMC10670808; doi:10.3390/gels9110868)
Supplement: Supplementary file 1 [file gels-09-00868-s001.zip › gels-2670362-supplementary.pdf]

Supplementary information:

Ultrasensitive Electroanalytical Detection of Pb^{2+} and H_2O_2 Using Bi and Fe—Based Nanoparticles Embedded into Porous Carbon Xerogel—The Influence of Nanocomposite Pyrolysis Temperatures

Mihai M. Rusu [1], Carmen I. Fort [2,3,*], Adriana Vulpoi [4], Lucian Barbu-Tudoran [5,6], Monica Baia [2,7], Liviu C. Cotet [2,3] and Lucian Baia [2,4,8,*]

1 Department of Physics and Chemistry, Technical University of Cluj-Napoca, 400114 Cluj-Napoca, Romania; mihai.rusu@phys.utcluj.ro
2 Laboratory of Advanced Materials and Applied Technologies, Institute of Research-Development-Innovation in Applied Natural Sciences, "Babes-Bolyai" University, Fantanele 30, 400294 Cluj-Napoca, Romania; monica.baia@ubbcluj.ro (M.B.); cosmin.cotet@ubbcluj.ro (L.C.C.)
3 Department of Chemical Engineering, Faculty of Chemistry and Chemical Engineering, "Babes-Bolyai" University, Arany Janos 11, 400028 Cluj-Napoca, Romania
4 Nanostructured Materials and Bio-Nano-Interfaces Center, Institute of Interdisciplinary Research in Bio-Nano-Sciences, "Babes-Bolyai" University, T. Laurean 42, 400271 Cluj-Napoca, Romania; adriana.vulpoi@ubbcluj.ro
5 Electron Microscopy Laboratory "Prof. C. Craciun", Faculty of Biology and Geology, "Babes-Bolyai" University, Clinicilor Str. 5–7, 400006 Cluj-Napoca, Romania; lucian.barbu@ubbcluj.ro
6 Electron Microscopy Integrated Laboratory, National Institute for Research and Development of Isotopic and Molecular Technologies, 400293 Cluj-Napoca, Romania
7 Department of Biomolecular Physics, Faculty of Physics, "Babes-Bolyai" University, M. Kogalniceanu 1, 400084 Cluj-Napoca, Romania
8 Department of Condensed Matter Physics and Advanced Technologies, Faculty of Physics, "Babes-Bolyai" University, M. Kogalniceanu 1, 400084 Cluj-Napoca, Romania
* Correspondence: ioana.fort@ubbcluj.ro (C.I.F.); lucian.baia@ubbcluj.ro (L.B.)

Table S1

A_{1g} Peaks

Sample	Position $_{D1}$	FWHM $_{D1}$	Position $_{D2}$	FWHM $_{D2}$
**No treatment**	1362	177	1256	182
**CXBiFe-600**	1364	149	1260	183
**CXBiFe-750**	1348	146	1260	190
**CXBiFe-900**	1352	176	1260	188
**CXBiFe-1050**	1352	179	1260	216
**CXBiFe-1050 (g)**	1342	60	1216	248

E_{2g} peaks

Sample	Position $_{G1}$	FWHM $_{G1}$	Position $_{G2}$	FWHM $_{G2}$
**No treatment**	1585	114	1532	223
**CXBiFe-600**	1595	60	1534	161
**CXBiFe-750**	1597	68	1523	178
**CXBiFe-900**	1601	63	1548	157
**CXBiFe-1050**	1601	60	1560	153
**CXBiFe-1050 (g)**	1575	42	1467	176

Relative intensities

Sample	I_{D1}/I_{G1}	I_{D2}/I_{G2}	I_{D2}/I_{G1}	I_{D2}/I_{D1}
**No treatment**	0.86	0.31	0.59	0.21
**CXBiFe-600**	0.72	0.81	0.33	0.37
**CXBiFe-750**	0.97	0.58	0.58	0.35
**CXBiFe-900**	1.46	0.30	0.78	0.16
**CXBiFe-1050**	1.70	0.16	0.90	0.08
**CXBiFe-1050 (g)**	0.92	0.73	0.10	0.08

The Raman peaks observed at ~1350 cm^{-1} and ~1600 cm^{-1} generally ascribed to the defect mediated double resonance D_1 band (A_{1g} breathing mode) and the G_1 band (E_{2g} mode) respectively, which is associated with the in-plane bond stretching vibrations for the sp^2 carbons found in aromatic and olefinic structures [22]. However, the spectra were best fitted using a system of four components (see **Table S1** for extracted parameters) as treated by others [45]. The additional peaks found at ~1290 and ~1540 cm^{-1} were interpreted as the D_2 and G_2 bands, reported in carbon materials with a higher level of disor-

der, due to fourfold coordination and bond angle disorder [45]. The Raman spectra from the Fe-graphitized regions were characterized by narrower D_1 and G_1 bands centered at 1341 and 1575 cm^{-1}, respectively, no significant D_2 and G_2 contributions and a fifth component denoted as G′. The G' band is centered around 1610 cm^{-1} and is associated with vibrations in the edges of graphitic crystallites, similar to graphites and carbon nanotubes, were found.

Table S2. Linear regression parameters for SWASV detection of Pb^{2+} at CXBiFe-T nanocomposites modified glassy carbon electrodes.

~~**Electrode type**~~	~~**Intercept (µA)**~~	~~**Slope (10^5 A/M)**~~	~~**R^2**~~	~~**N**~~
~~**GC/Chi-CXBiFe-600**~~	~~1.78 ± 0.04~~	~~0.20 ± 0.07~~	~~0.9921~~	~~7~~
~~**GC/Chi-CXBiFe-750**~~	~~0.63 ± 0.09~~	~~0.49 ± 0.01~~	~~0.99352~~	~~11~~
~~**GC/Chi-CXBiFe-900**~~	~~1.35 ± 0.02~~	~~2.52 ± 0.42~~	~~0.99726~~	~~11~~
~~**GC/Chi-CXBiFe-1050**~~	~~1.30 ± 0.01~~	~~7.01 ± 0.71~~	~~0.99908~~	~~10~~

**Electrode type**	**Intercept (µA)**	**Slope (10^5 A/M)**	**R^2**	**N**
**GC/Chi-CXBiFe-600**	1.78 ± 0.04	0.20 ± 0.07	0.9921	7
**GC/Chi-CXBiFe-750**	0.63 ± 0.09	0.49 ± 0.01	0.99352	11
**GC/Chi-CXBiFe-900**	1.35 ± 0.02	2.52 ± 0.42	0.99726	11
**GC/Chi-CXBiFe-1050**	0.45 ± 0.03	9.22 ± 0.33	0.98691	11

Table S3. Repetability of Pb^{2+} detection at GC/Chi-(CXBiFe-1050) modified electrodes.

Analyte	Peak potential*		Peak current*	
	V *vs.* Ag/AgCl,KCl_{sat}	RSD (%)	µA	RSD (%)
9 pM Pb^{2+}	-0.401 ± 0.004	1.15	8.146 ± 0.05	0.618

*Results obtained as averages for 4 different electrodes.

Table S4. SWASV recorded at GC/Chi-(CXBiFe-1050) and AAS results for Pb^{2+} determination in real samples of drilling water.

	AAS* (pM)	Measured Pb^{2+}(pM)	Relative error (%)	RSD (%)
**Sample 1**	3.85	3.92 ± 0.06**	+1.8	1.34
**Sample 2**	4.85	5.09 ± 0.19**	+4.8	2.98

*Atomic absorption spectroscopy

** The average of peak current resulted from three different measurements.

Pyrolysis induced changes at micro- and nanometric scales

Cracks and other microscale defects (see **Figure S1a**) along with the signaled pyrolysis induced changes over the pore network are expected to influence the mechanical properties of the nanocomposites and indirectly, the CXBiFe-T grain size during grinding under constant conditions. This is confirmed through SEM analysis of the ground samples (**Figure S1b**) and DLS measurements (see **Figure S**2).

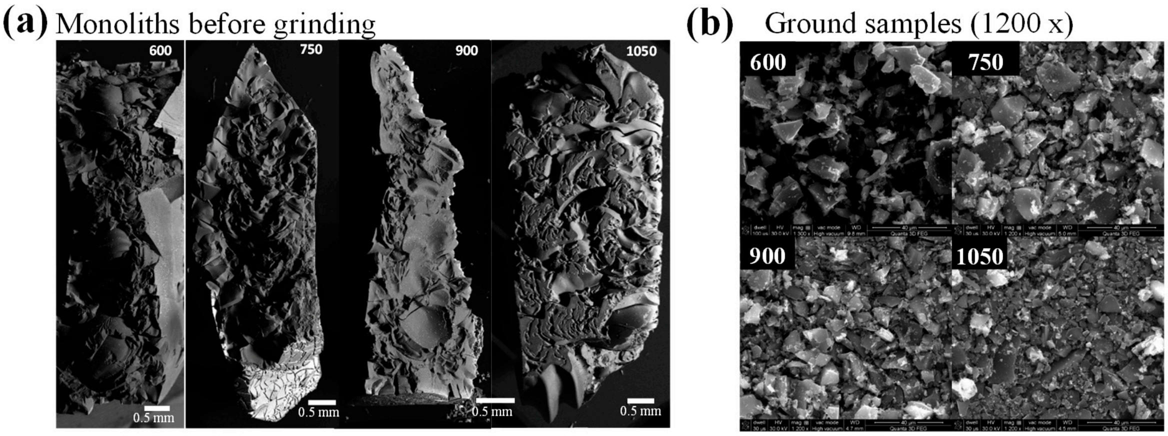

Figure S1. CXBiFe-T samples at microscopic and nano-scales during SEM analysis **(a)** reconstructed wide range images of the CXBiFe-T monoliths, **(b)** SEM micrographs at 1200 x of CXBiFe-T powders after grinding

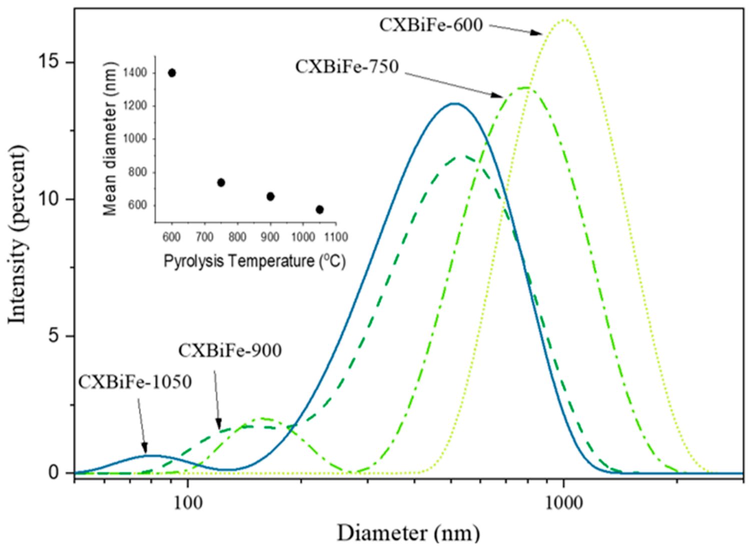

Figure S2. DLS measurements showing the size distributions of CXBiFe-T granules smaller than 5 µm.

Through DLS measurements performed on the CXBiFe-T samples after controlled grinding, a decrease is observed from an average D_{DLS} of 1.40 µm (for CXBiFe-600) to 0.57 µm (for CXBiFe-1050). Also, the shape of the granule size distribution is modified by a second contribution of smaller particles found in the range of 50-300 nm for the samples treated above 750°C.

With respect to the distribution of Bi and Fe phases into the nanocomposite, different regions from the monoliths were inspected and classified as carbonaceous granules with embedded metal/oxide nanoparticles as dominant feature (**Figure S3**), scattered micro-particles originating from the external crust (**Figure S4**) and Bi spikes formed at granule boundaries (**Figure S5**). The elemental analysis of the dominant feature, the carbonaceous granules indicates a tendency of gradual decrease in oxygen percentage from a total of 25.5 at%, for the prepared sample, to an average of 4.8 at%, for the sample CXBiFe-1050. A decrease in the bismuth fraction is also observed. The presented data is consistent with the general XRD results.

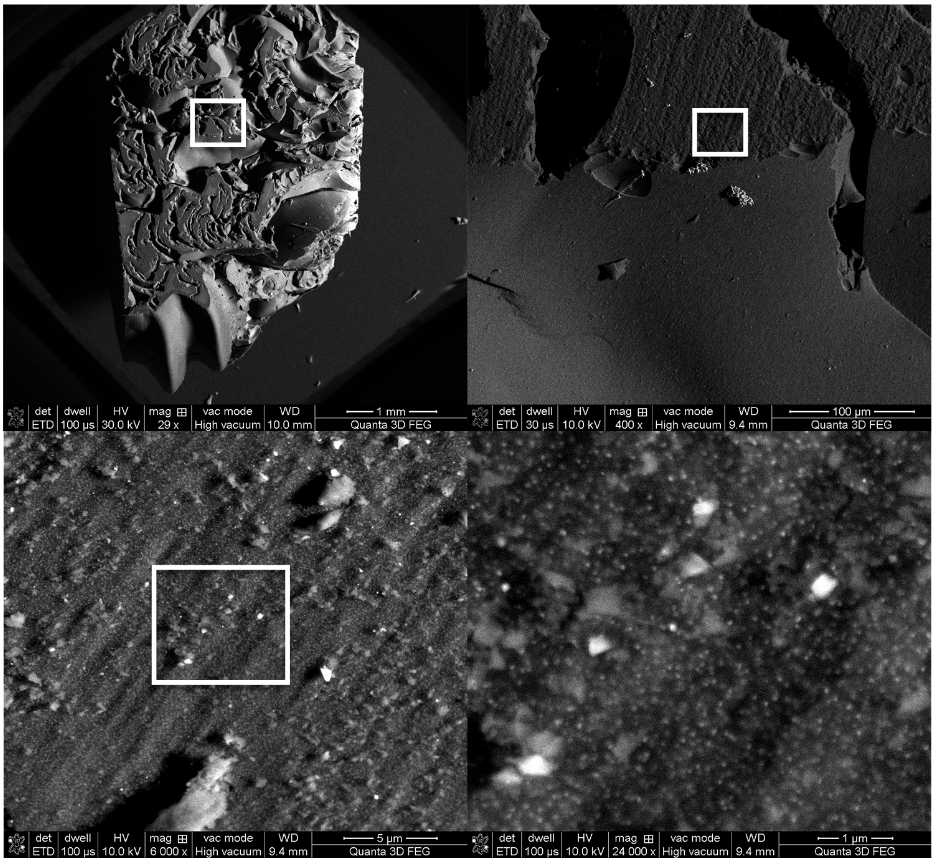

Figure S3. The main features observed for CXBiFe-T nanocomposites: carbon granules with embedded nanoparticles

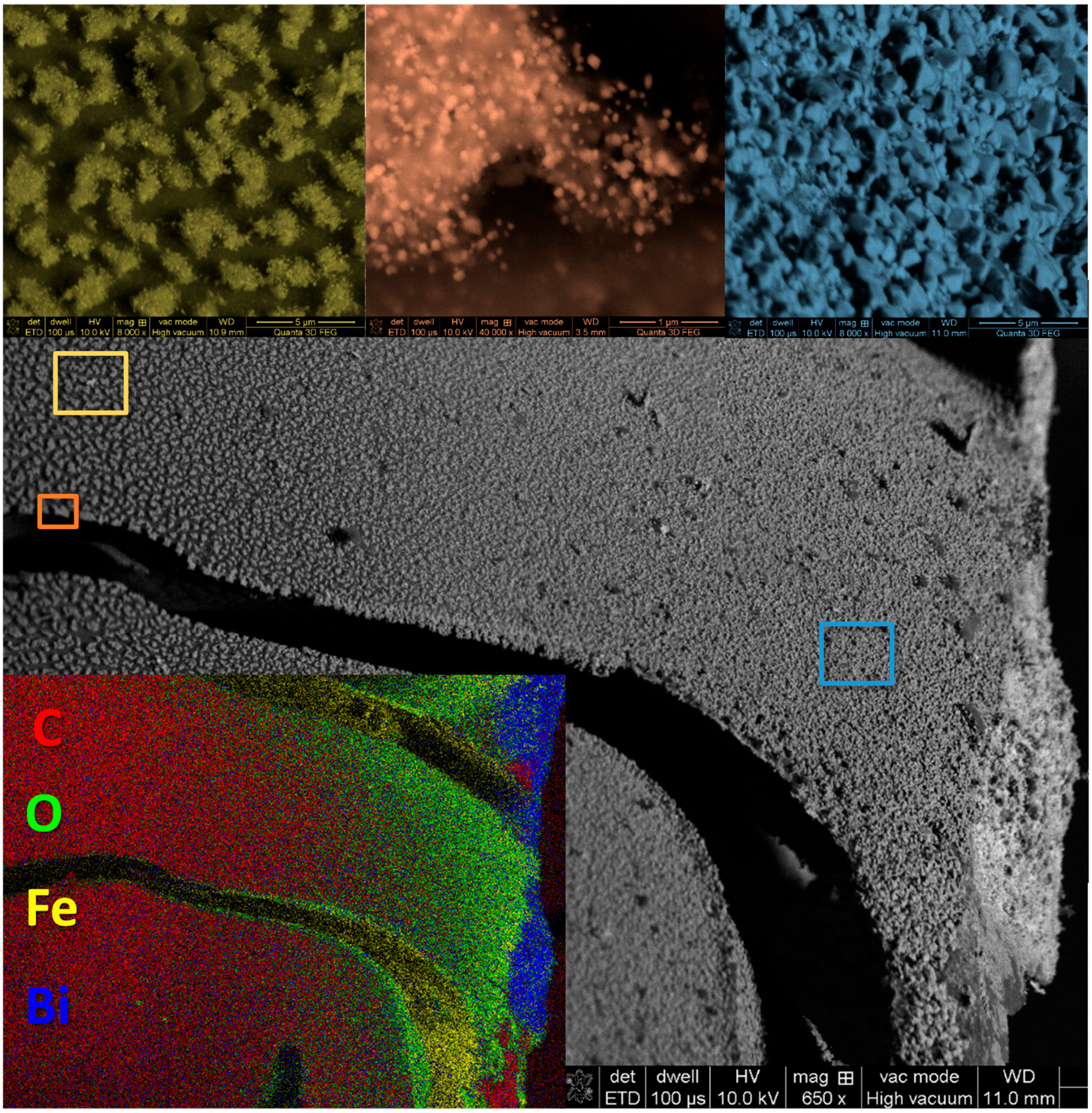

Figure S4. Formation of oxidic crust towards the surface of CXBiFe-T nanocomposites

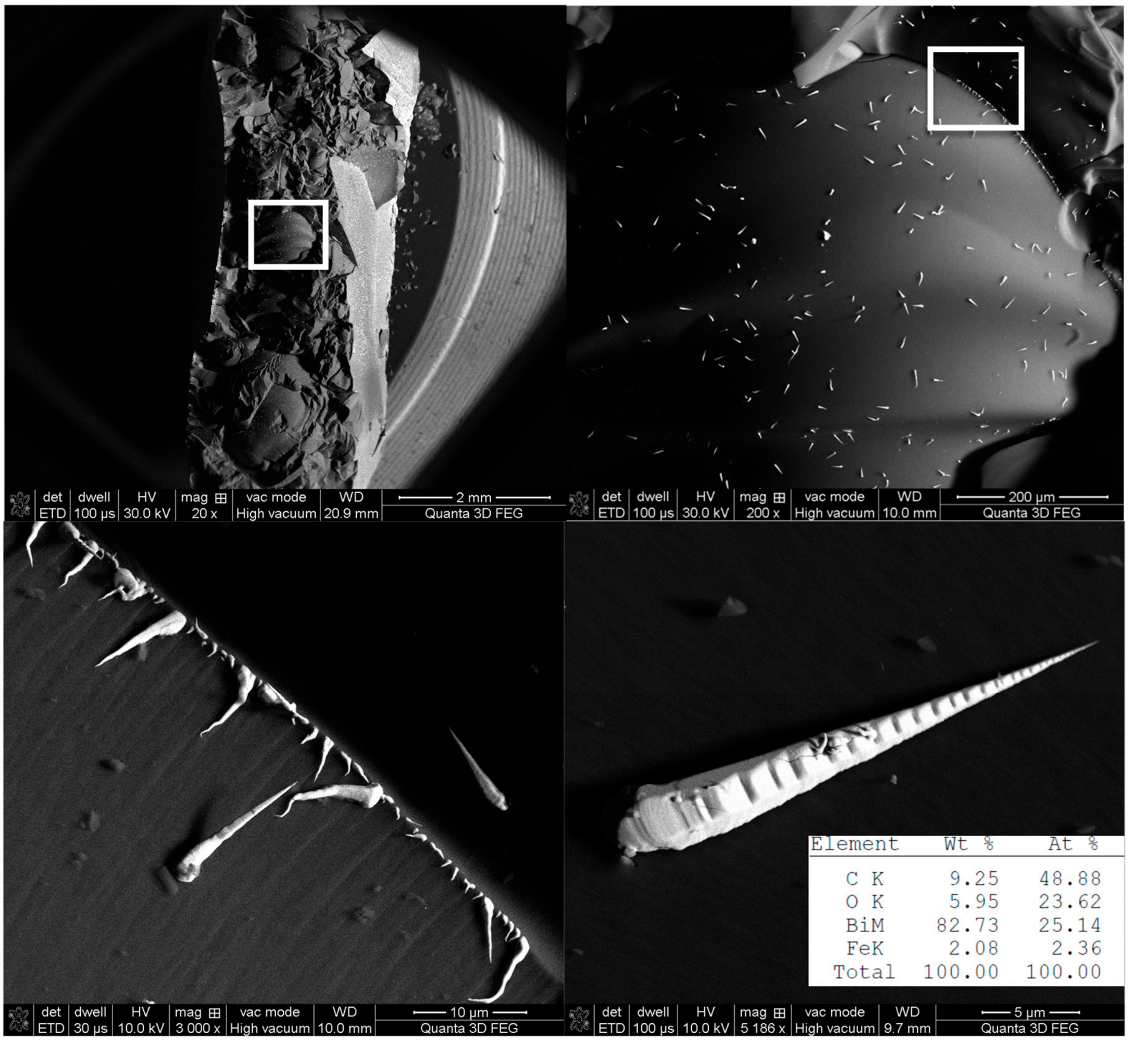

Figure S5. Micron size spike formation at surface, cracks and micro-cavities in CXBiFe-T nano-composites.

Micron-sized spikes were detected when probing fractured plane of the monoliths as shown in **Figure** S5. The quantification of the EDX spectra gathered from the micro-spikes indicate that their composition is mostly based on Bi. It is expected that, due to Bi liquefaction at high temperatures, particles start to coalesce and develop large micro-structures at the interface with macro-cavities and cracks. If the temperature drops during their migration towards energetically more favorable states, the liquid drops solidify forming the observed micron sized spikes.

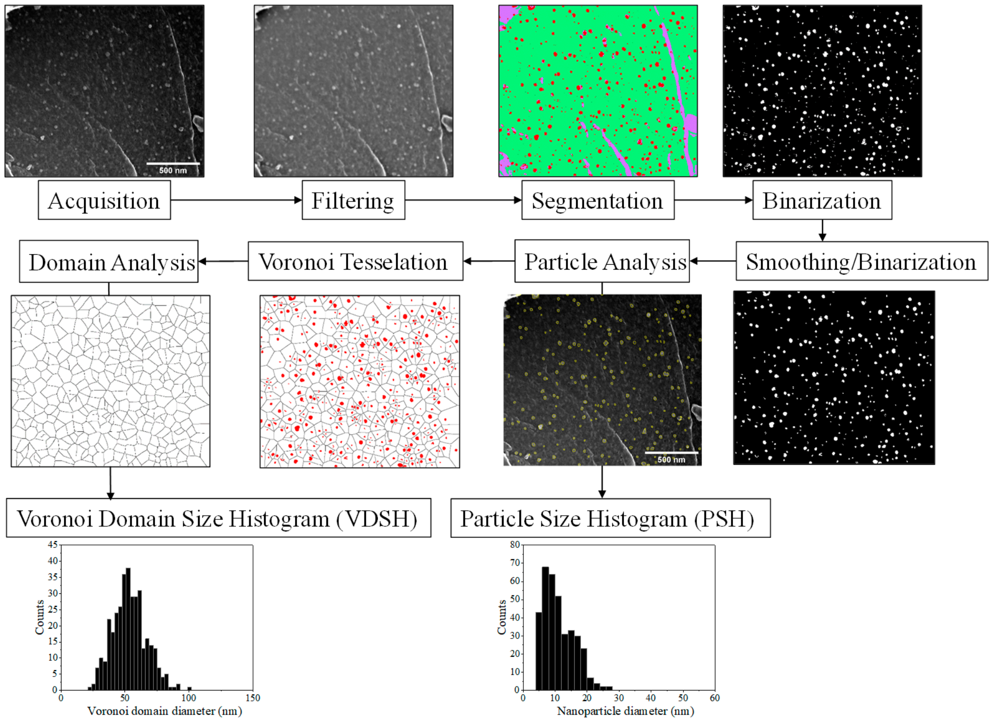

Figure S6. Image processing sequence for PSH and VDSH determination from SEM micrographs

First, the SEM micrographs of flat grain regions were acquired at magnifications of 24000-80000 x. Prior to image segmentation, a band pass filter was applied on the Fourier transform in order to reduce noise and keep only the nanoparticle related 5-75 px details. For autonomous particle discrimination from the surrouding carbon support and from imperfections such as crests pores or deposits, the images were segmented using the Trainable Weka Segmentation v3.2.34 plugin [48]. After binarization, the resulting features with areas larger than 100 nm^2 and with a circularity factor greater than 0.3 were analyzed to obtain the “nanoparticle area” and for tesselation, the coordinates of the center of mass for each individual particles. Better results in terms of noise level and predicted particle size were obtained when the binarized images were further smoothed and binarized again. Voronoi tesselation was performed to divide the inter-particle space into cells reffered to as Voronoi domains for each of the detected nanoparticles. Then, the area of the Voronoi domains was measured. The particle size histograms (PSH) and Voronoi domain size historgrams (VDSH) were constructed by using the diameters of disks having the areas equal to each particle or domain area.

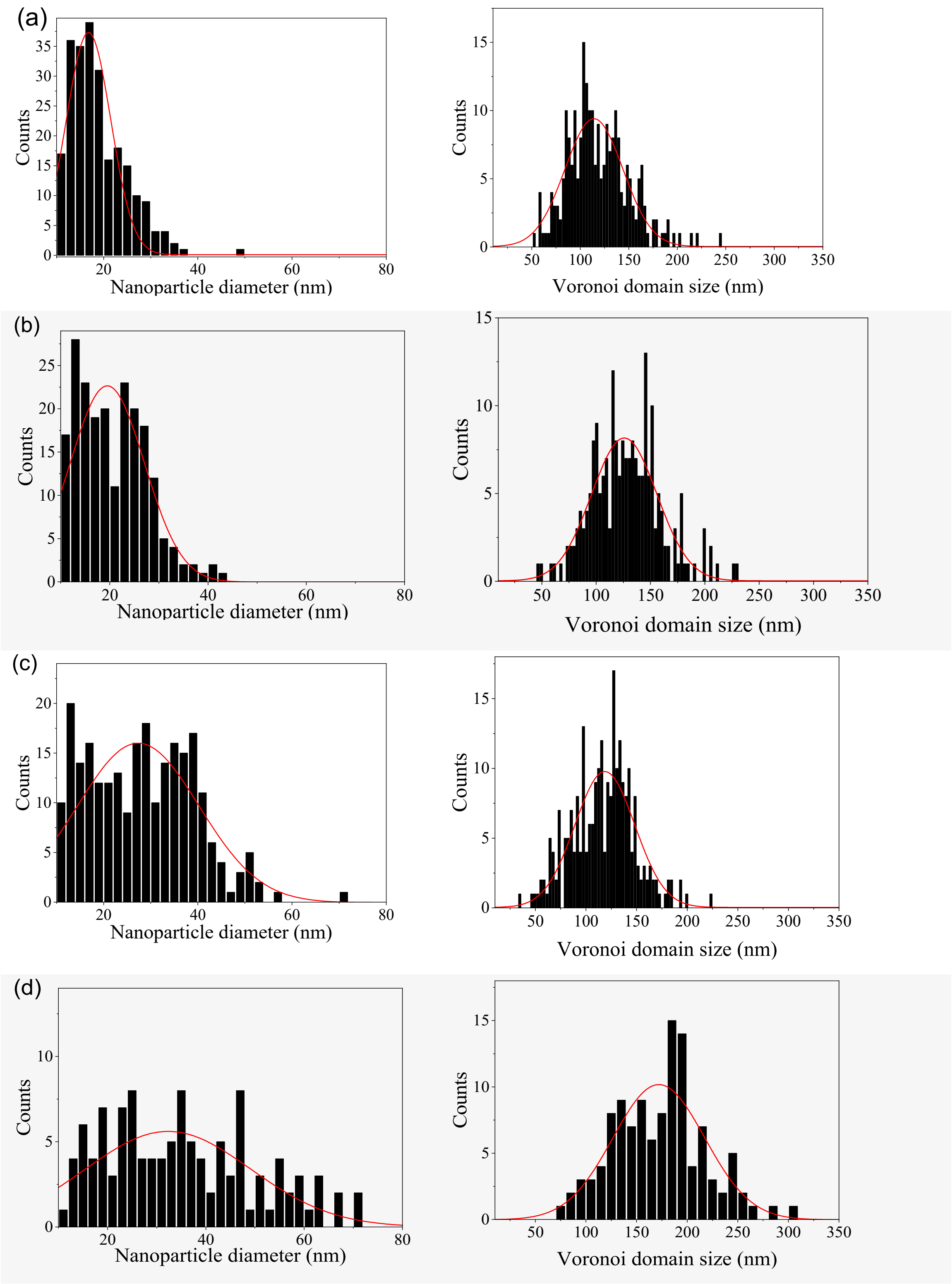

Figure S7. PSH and VDSH obtained from SEM micrographs from the surface of CXBiFe: (a) 600, (b) 750, (c) 900 and (d) 1050.

Figure S8.

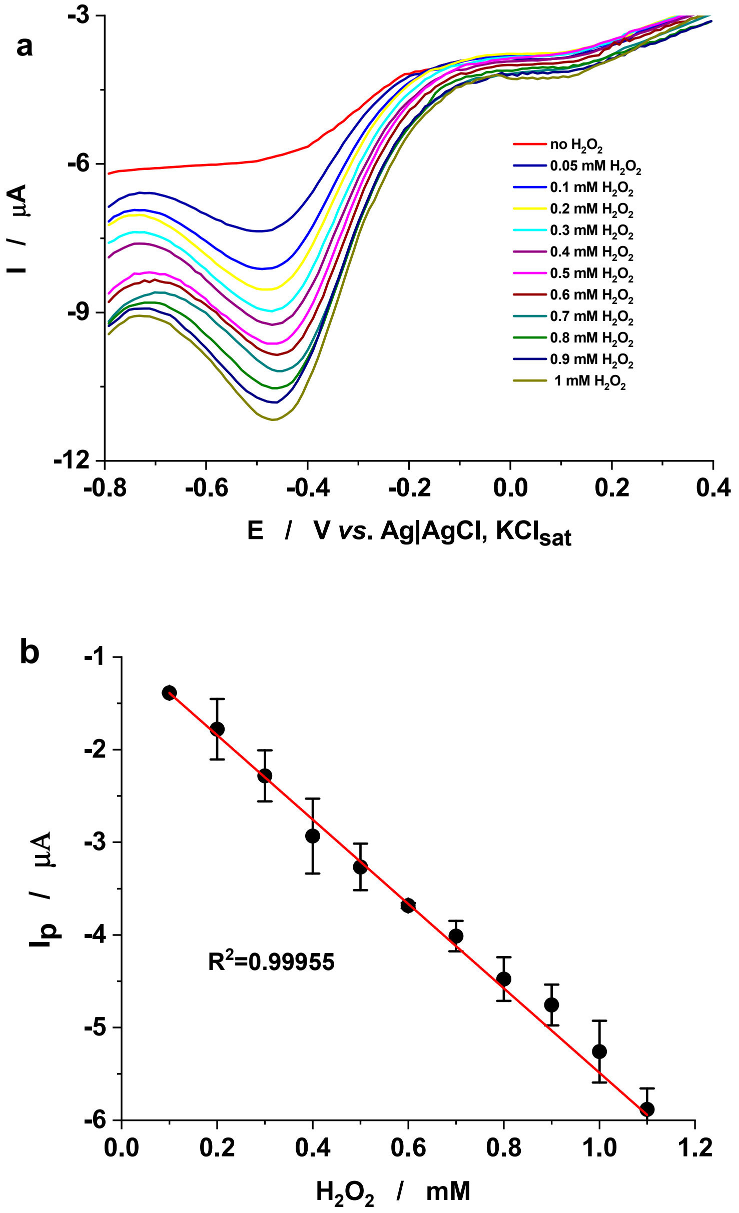

Figure S8. (a) SW voltammograms recorded at GC/Chi-(CXBiFe-1050) electrodes in presence of different concentrations of H_2O_2. Experimental conditions: supporting electrolyte, 0.1 M phosphate buffer (pH 7); frequency, 10 Hz; amplitude, 25 mV. (b) The corresponding calibration curve for H_2O_2 detection.

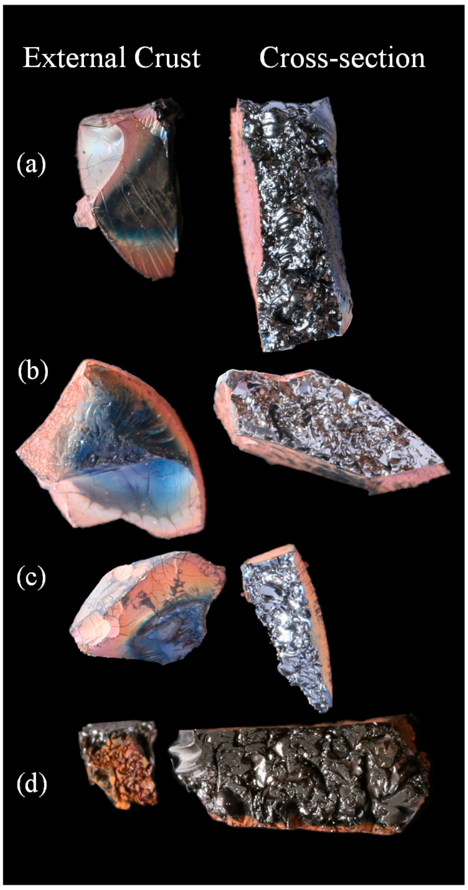

Figure S9. Optical images of CXBiFe monoliths after pyrolysis at (a) 600, (b) 750, (c) 900, (d) 1050 °C.

Optical images of the monoliths taken from the sample surface and cross-section are shown in **Figure S**9. The monoliths exhibit a cracked and crusty surface with colors ranging from iridescent-blue (mostly attributed to Bi deposits) to rusty orange (attributed to Fe_3O_4 aggregates). The core of the monoliths exhibits a homogeneous dark-metallic aspect attributed to the carbon component as dominant phase. The shape of the fractured surfaces before and after polishing also indicate the presence of macroscopic pores and cavities.
